# Carbon-ion radiotherapy for non-small cell lung cancer with interstitial lung disease: a retrospective analysis

**DOI:** 10.1186/s13014-017-0881-1

**Published:** 2017-09-02

**Authors:** Mio Nakajima, Naoyoshi Yamamoto, Kazuhiko Hayashi, Masataka Karube, Daniel K Ebner, Wataru Takahashi, Makoto Anzai, Kenji Tsushima, Yuji Tada, Koichiro Tatsumi, Tadaaiki Miyamoto, Hiroshi Tsuji, Takehiko Fujisawa, Tadashi Kamada

**Affiliations:** 1 National Institute of Radiological Sciences Hospital, National Institutes for Quantum and Radiological Sciences and Technology, 4-9-1, Anagawa, Inage-ward, Chiba, 263-8555 Japan; 2 Department of Radiology, the University of Tokyo Hospital, Hongo, Bunkyo-ward, Tokyo, 113-8655 Japan; 3 Brown University Alpert Medical School, Providence, RI 02903 USA; 4 Kansai Rosai Hospital, Inabaso, Amagasaki, 660-8511 Japan; 5 Department of Respirology, Graduate School of Medicine, Chiba University, Inohana, Chuo-ward, Chiba, 260-8670 Japan; 6 Chiba Foundation for Health Promotion and Disease Prevention, Shinminato, Mihama-ward, Chiba, 261-0002 Japan

**Keywords:** Carbon ion radiotherapy, Lung cancer, Interstitial lung disease, Radiotherapy, Radiation pneumonitis

## Abstract

**Background:**

Lung cancer is frequently complicated by interstitial lung disease (ILD). Treatment protocols for lung cancer patients with ILD have not been established; surgery, chemotherapy, and radiotherapy can all cause acute exacerbation of ILD. This study evaluated the toxicity and efficacy of carbon ion radiotherapy (CIRT) in patients with non-small cell lung cancer (NSCLC) and ILD.

**Methods:**

Between June 2004 and November 2014, 29 patients diagnosed with NSCLC and ILD were treated with CIRT. No patient was eligible for curative surgery or conventional radiotherapy secondary to ILD. Owing to prior symptomology, radiation pneumonitis (RP) and symptom progression pre- and post-treatment were evaluated. The relationships between RP and clinical factors were investigated.

**Results:**

Twenty-eight men and one woman, aged 62 to 90 years old, were followed for 2.7–77.1 months (median: 22.8 months). Single-grade symptomatic progression (grade 2–3) was observed in 4 patients, while 1 patient experiencedtwo-grade progression. Two patients experienced radiation-induced acute exacerbation. Local control at 3 years was 63.3% (72.2% for stage I disease); survival at 3 years was 46.3% (57.2% for stage I disease). Eighteen patients had died by the time of this writing, 10 of lung cancer progression. Radiation pneumonitis post-treatment progression correlated with dosimetric factors of the lungs (V5, V10) and a low pre-treatment serum surfactant protein-D.

**Conclusions:**

We found that CIRT may be useful as a low-risk, curative option for NSCLC patients with ILD, a population that is typically ineligible for conventional therapy. The DVH analysis showed that minimizing the low-dose region is important for reducing the risk of severe RP.

**Trial registration:**

NIRS-9404. Registered 1 March 1994.

## Background

Lung cancer (LC) is often associated with interstitial lung disease (ILD). The reported prevalence of LC in ILDs is 13–50%, particularly in the setting of interstitial pulmonary fibrosis (IPF) [1–4].

Treatment protocols for ILD-associated LC have not been well-established, as surgery, chemotherapy, and radiotherapy can all cause acute exacerbation (AE) of ILD. The postoperative AE frequency has been reported to be 9–25%; with a mortality rate of 11–86%, it accounts for the majority of treatment-related deaths [5–9].

Stereotactic body radiation therapy (SBRT) is a noninvasive cancer treatment, and has proven to be an effective and well-tolerated treatment in numerous studies for early stage LC in medically inoperable patients [10–12]. However, severe ILD is considered a relative contraindication in the clinical guidelines for SBRT published by the Japanese Society for Therapeutic Radiation and Oncology, as a Japanese national survey of SBRT found that most patients with pulmonary grade 5 radiation pneumonitis (RP) had ILD [13]. High post-treatment mortality, even with mild ILD, has caused great difficulty in designing treatments for ILD-LC patients, and treatment results remain unsatisfactory [14].

In 1994, the National Institute of Radiological Sciences (NIRS) began exploring carbon-ion radiotherapy (CIRT) as a treatment for non-small cell lung cancer (NSCLC). Carbon-ion beams offer unique treatment advantages compared to other radiation modalities, including the delivery of a highly precise dose concentration to the target, with high linear energy transfer and relative biological effectiveness throughout the spread-out Bragg peak [15]. As reported previously, hypofractionated CIRT has produced excellent local control rates that are comparable to those of surgery or SBRT, with markedly low toxicities [16–19].

In this study, we evaluated the toxicity and efficacy of CIRT for treating NSCLC in patients also presenting with ILD.

## Methods

### Study design and patient selection

We performed a retrospective evaluation of all patients with ILD-LC who were treated with CIRT at our hospital. Owing to the presense of ILD, all enrolled patients were ineligible for curative surgery and conventional radiotherapy.We diagnosed ILD was according to medical history, physical examination, respiratory function test, and evaluation of abnormalities compatible with bilateral lung fibrosis on chest computed tomography (CT) or high-resolution CT, such as peripheral reticular opacities. Biochemical tests for levels of lactate dehydrogenase, surfactant protein-D (SP-D), and serum Krebs von den Lungen-6 (KL-6) were also performed.

The treatment method and procedure were approved by the ethics committees of our institute, and written informed consent was obtained from all patients included in the study.

### Lung cancer staging and interstitial lung disease severity

Initial workup included clinical and laboratory examinations, contrast-enhanced CT of the chest, contrast-enhanced magnetic resonance imaging (MRI) of the brain, and [11C]-acetate or [18F]-fluorodeoxyglucose (^18^F–FDG) positron emission tomography (PET)/CT scanning for detecting involved lymph nodes and distant metastases. The severity of ILD was evaluated according to the criteria of the Japanese Respiratory Society classifications [20].

### Carbon-ion radiotherapy procedure

Carbon ion beams with 290, 350, and 400 MeV of nucleon energy were generated in the Heavy Ion Medical Accelerator in Chiba (HIMAC) synchrotron and delivered to the treatment room. The details of CIRT planning and delivery at our institution have been described previously [16, 18, 19, 21].

Between June 2004 and November 2014, CIRT was performed on 637 patients with primary NSCLC lesions at our institution; among them, 29 patients had ILD. Two patients were diagnosed with hilar lymph node metastasis. Three patients had suspected mediastinal lymph node metastasis on PET/CT, and 1 had suspected accessory nerve lymph node metastasis. We treated only the primary lesions of these patients, as Konishi et al. and Roberts et al. reported that inflammatory pulmonary lesions may show increased uptake of 18F–FDG [22, 23]. Additionally, 1 patient was diagnosed with an intrapulmonary metastatic lesion near their primary tumor. Both primary and metastatic lesions were treated in the same field.

For stage I LC patients and 4 patients with suspected lymph node metastases, the primary tumors were irradiated with carbon-ion beams at a fixed dose of 52.8 Gy (relative biological effectiveness [RBE]) (13.2 Gy[RBE]/fraction) for T1 (≤3 cm) tumors and 60.0 Gy (RBE) (15 Gy[RBE]/fraction) for T2 (>3 cm) tumors, delivered in 4 fractions over the course of 1 week. A single T4 patient was included in the T2 tumor group; this patient’s primary tumor and nearby intrapulmonary metastatic lesion were irradiated. After February 2012, primary tumors in stage I LC patients were irradiated in a single fraction at doses prescribed by the NIRS Lung Cancer Single Fraction Clinical Trial [19]. For single fraction delivery, tumors were normally irradiated from 4 ports consecutively. The HIMAC delivery system allowed for patients to be rotated a maximum of ±20 degrees, allowing for four different treatment angles between the horizontal and vertical particle beams.

In accordance with routine practice at our institute for stage I NSCLC, all CIRT plans utilized 4 coplanar and oblique beam fields at mutual angles of 40 or 50 degrees.

For two patients with stage II NSCLC who had hilar lymph node metastases, the primary tumor and involved lymph nodes were contoured as the gross tumor volume. The clinical target volume included the primary tumor with 10-mm margins in all directions. Prophylactic regional nodal irradiation that included targeting the ipsilateral hilar and mediastinal lymph nodes was chosen for one of the patients with a hilar lymph node metastasis. For the other patient, only the primary tumor and involved lymph node were irradiated. The prescribed dose (72 Gy [RBE]) was administered to the primary tumor, and a reduced dose was delivered to the CTV up to a total dose of 50 Gy (RBE) at the discretion of the treating physician. A fixed dose of 72 Gy (RBE) was delivered over a course of 16 daily fractions administered on four consecutive days per week and over four consecutive weeks.

**Fig. 1:**
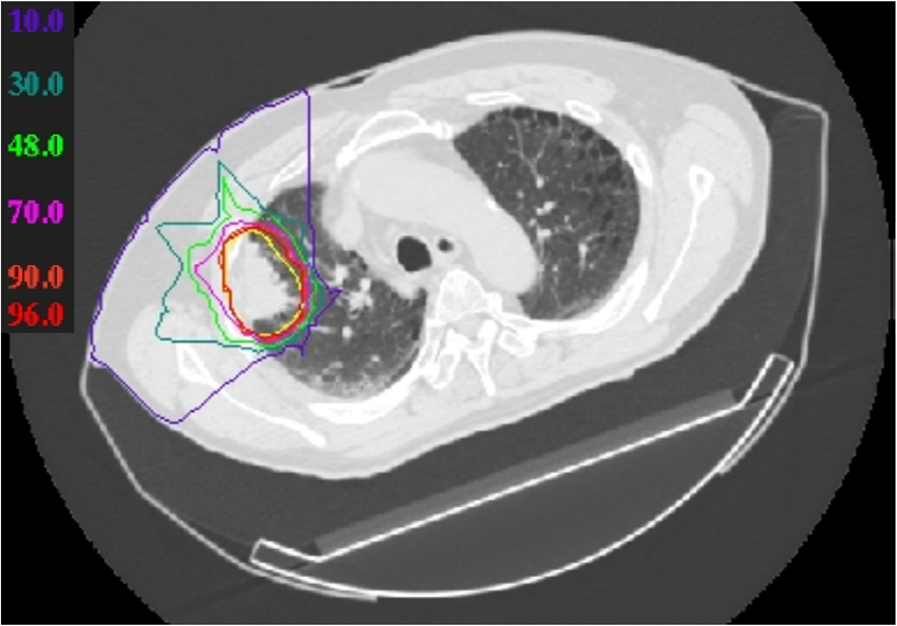
Carbon-ion dose distribution in a patient with stage I non–small cell lung cancer. The yellow and red lines indicate clinical target volume and 95% isodose of the prescribed dose, respectively

The planning target volume (PTV) was enclosed by the 95% isodose line for conformal treatment with the prescribed dose. Dose constraints were strictly adhered to, regardless of potential target coverage compromise, as follows: spinal cord, 30 Gy (RBE); esophagus, 50 Gy (RBE); and mainstem bronchus, 60 Gy (RBE). Figure 1 shows a sample of the dose distribution for a patient wih stage I disease.

### Follow-up

After the end of radiotherapy, regular follow-up examinations were performed every 3 months for the first 2 years and every 6 months thereafter. Each follow-up session included a physical evaluation, chest CT, brain MRI, respiratory function test, and laboratory tests at a minimum. Additional imaging investigations such as bone scanning and PET/CT were performed upon clinical suspicion of recurrence.

### Radiation pneumonitis grading and the definition of acute exacerbation

The severity of RP was graded according to National Cancer Institute Common Terminology Criteria for Adverse Events (CTCAE), version 3.0. As many patients had respiratory symptoms prior to CIRT, symptoms were graded before and after CIRT, and symptomatic grade change was evaluated. The occurrence of AE was defined according to the revised Japanese criteria for AE of IPF [20], which states that all of the following three conditions must be satisfied during the course of IPF within a single month: 1) worsening or development of dyspnea 2) new ground-glass opacities appear on high-resolution CT in addition to previous honeycomb lesions, new ground-glass abnornalities, and/or consolidation superimposed on a background reticular or honeycomb pattern on high-resolution CT 3) a greater than 10 mmHg reduction of partial pressure of oxygen in the arterial blood under similar conditions. The ruling out of alternative causes, such as infection, pneumothorax, cancer, pulmonary embolism or congestive heart failure is required. Due to the difficulty of distinguishing AE from RP, AE was confirmed when infiltrates were observed outside the radiation field.

### Statistical analysis

All data are presented as median values with ranges. We used JMP statistical software (version 11.0) for all statistical analyses. To investigate the relationships between patients with or without IP grade progression, clinical characteristics and treatment-related factors were compared using the Mann-Whitney U test or Fisher’s exact test.

Overall survival was defined as the time elapsed from the start of CIRT to death or to last follow-up date (i.e., last hospital visit, phone call, or mailed communication). Patients lost to follow-up or who were alive at the end of the study were censored. The local control rate was calculated from the date of initial CIRT to the first local recurrence was first detected, or that of the patient’s last follow-up visit. The overall survival and local control rates were analyzed using Kaplan-Meier statistics. Cox proportional hazards analysis was conducted to identify prognostic factors. A *p*-value less than 0.05 was considered statistically significant. Any AEs occurring after radiotherapy were evaluated qualitatively on an individual basis due to their low numbers.

## Results

### Patient characteristics and the severity of interstitial lung disease

In total, 637 consecutive patients with NSCLC who underwent CIRT between June 2004 and November 2014 were investigated retrospectively. Of these, 29 patients with ILD were identified; their characteristics are listed in Table 1. Prior to CIRT, 11 grade 1 patients, 6 grade 2 patients, 8 grade 3 patients, and 4 grade 4 patients were identified according to the Modified Medical Research Council Dyspnea Scale [24]. Under the CTCAE v.3.0 criteria, there were 11 grade 1 patients, 14 grade 2 patients, 4 grade 3 patients, and no grade 4 or 5 patients. Four patients were using home oxygen therapy, 4 had a history of AE prior to treatment, and 4 had a prior autoimmune disease.

**Table 1:** Pretreatment patient characteristics

No. of patients	29
Follow-up period median (range)	26.8 (2.7–86.5)
Gender (male/female)	28/1
Age median (range)	73 (62–90)
Performance Status (ECOG) 0/1/2/3	15/13/2/0
Smoking history (pack years) median (range)	42 (0–210)
Severity of ILD (grade 1/2/3/4)	9/10/6/4
Dyspnea evaluation before CIRT	
No. of patients using home oxygen therapy	4
mMRC scale (grade 0/1/2/3/4)	0/11/6/8/4
equivalent RP grade CTCAE ver 3.0 (grade 1/2/3/4/5)	11/14/4/0/0
%FVC median (range)^a^	82.3 (37.9–117.6)
%DLco median (range)^a^	52.1 (15.5–235.5)
Tumor size	34 (17–63)
T1a (≤2 cm)	2
T1b (2-3 cm)	8
T2a (3-5 cm)	14
T2b (≥5 cm)	5
Tumor location	3000
Upper lobe (including 2 tumors in middle lobe)	15
Lower lobe	14
Nodal involvement (yes/no/suspected)	2/23/4
Location of nodal involvement of 2 patients	#11 (left hilar lymph node)
Histology	
Adenocarcinoma	8
Squamous cell carcinoma	10
Unclassified non-small cell carcinoma	2
Clinically diagnosed	9
Laboratory Data	
serum KL-6 (U/l) median (range)	1167 (456–2410)
serum SP-D (ng/l) median (range)	157 (31–502)
The time of ILD diagnosis	
at the same time as lung cancer diagnosis	14
unknown	1
before diagnosis of lung cancer	14
Period from ILD to cancer diagnosis (median years) (range)	4 (2–13)
Episode of acute exacerbation before treatment (yes/no)	4/25
Auto-immune disease (yes/no)	4/25

*data are available only for 26 patients

*Abbreviations*: *ECOG* Eastern Cooperative Oncology Group, *ILD* interstitial lung disease, *CIRT* carbon ion radiotherapy, *mMRC scale* the modified Medical Research Council scale, *FVC* forced vital capacity, *DLco* diffusion capacity of the lung, *KL-6* Krebs von den Lungen-6, *SP-D* surfactant protein D

### Treatment characteristics

An evolving series of protocols were employed during the treatment period, the details of which are shown in Table 2. Treatment timeframes ranged between 1 day (46–50 Gy [RBE], single fraction; 13 patients), 4 days (52.8–60 Gy[RBE], 4 fractions; 14 patients), and 4 weeks (72 Gy[RBE], 16 fractions; 2 patients). The PTV ranged from 34.6 mL to 1319.8 mL (median: 145.1 mL).

**Table 2:** Treatment characteristics and dose volume analysis

Protocol	No. of pts.
46.0Gy (RBE) /1fr/1 day	1
48.0Gy (RBE)/1fr/1 day	4
50.0Gy (RBE)/1fr/1 day	8
52.8Gy (RBE)/4fr/4 days	7
60.0Gy (RBE)/4fr/4 days	7
72.0Gy (RBE)/16fr/4wks	2
PTV volume (ml), median (range)	85.3 (34.6–1319.8)
Dosimetric factors of the lung (%), median (range)	
V5 lung	10.5 (3.0–34.8)
V10 lung	8.6 (2.6–32.8)
V15 lung	7.1 (2.2–27.1)
V20 lung	6.2 (2.0–18.0)
V25 lung	4.6 (1.8–16.8)
V30lung	4.0 (1.5–15.9)
Mean Lung Dose	3.5 (1.0–12.2)

*Abbreviations*: *RBE* relative biological effectiveness, *PTV* planning target volume

### Pre- and post-treatment radiation pneumonitis and radiation-induced AE

We detected RP of grades 1, 2, and 3 in 9, 12, and 8 patients, respectively (Table. 3). Five patients experienced RP grade progression. Four patients experienced a single RP grade increase, from 1 to 2 or 2 to 3, following treatment; 1 patient had a 2-degree increase, from 1 to 3. Two patients experienced radiation-induced AE.

**Table 3:** RP Grade and incidence of acute exacerbation

RP grade (CTCAE ver3.0)	
1	9 (31%)
2	12 (41%)
3	8 (28%)
RP grade progression after treatment	
0/1/2	24/4/1
Acute exacerbation	2 (7%)^a^
RP symptom onset time	
no symptom	15 (52%)
0–3 month	9 (31%)
3–6 month	5 (17%)

^a^These patients are also included in RP grade 3 data

*Abbreviations*: *CTCAE* Common Terminology Criteria for Adverse Events, *RP* radiation pneumonitis

### Clinical outcomes

**Fig. 2:**
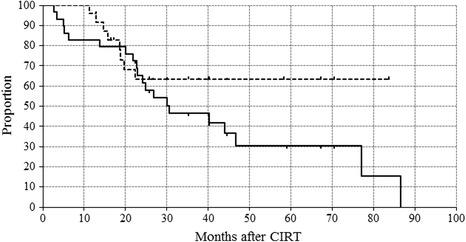
Overall survival (OS) and local control rates (LCR) after carbon-ion radiotherapy. The solid line is the OS, and the dotted line is the LCR in 29 patients. The 3- and 5 year LCRs were both 63.3% and the 3- and 5 year OS were 46.3% and 30.4% respectively

The median follow-up period was 26.8 months (range, 2.7–86.5 months) for all patients. The overall survival rates at 3 and 5 years was 46.3 and 30.4% (57.2 and 42.4% for stage I disease), respectively (Fig. 2). Eighteen of 29 patients (62.1%) showed a survival of longer than 2 years. Eighteen patients had died at the time of this writing, 10 of LC progression, 2 of other cancers (1 pancreastic, and 1 myelodysplastic syndrome), and 6 of noncancerous causes (3 of bacterial pneumonia, 2 of hypoxia due to progression of ILD, and 1 of gastrointestinal hemorrhage). The deaths from hypoxia resulting from ILD occurred more than 40 months after undergoing CIRT in both patients. We observed eight local recurrences; the median time between CIRT and diagnosis of local recurrence was 17.2 months. The local control rates at 3 and 5 years were both 63.3% (72.2% for stage I disease only).

### Acute exacerbation

Two patients were diagnosed with AE of their ILD post-CIRT. One was a 76-year-old man with stage IA LC, and the other an 80-year-old man with stage IB LC. They were diagnosed with AE by CT scanning following hospital admission due to worsening dyspnea, which revealed ground glass opacities outside the irradiated field. Both were treated with oxygen and steroid pulse therapy. The AE subsided in both patients, although the man with stage IA disease died of gastrointestinal hemorrhage 1 month following admission. The patient with stage IB disease was discharged, but died of bacterial pneumonitis 5 months after radiotherapy.

### Radiation pneumonitis grade progression and related factors

Table 4 shows the relationships between the RP grade progression and pretreatment clinical factors as well as dosimetric factors in the 29 patients. The lung doses of V5 and V10 were significantly associated with the occurrence of RP grade progression (*p =* 0.026 and 0.033). Pre-treatment serum SP-D was also found to correlate with grade progression, and was significantly lower in the grade-progressing patient population (*p =* 0.036).

**Table 4:** Clinical and dosimetric factors associated with symptoms after CIRT

	no grade change (*n* = 24)	grade progression (*n* = 5)	*p*-value
Clinical factors			
Age (yr), median (range)	72.0 (62–90)	76.0 (71–86)	0.224
Performance Status (0/1/2)	13/9/2	1/4/0	0.330
Severity of ILD (JRS) (1/2/3/4)	9/7/4/4	0/3/2/0	0.434
mMRC scale (1/2/3/4/5)	0/9/6/5/4	0/2/0/3/0	0.952
Tumor location (U/L)	11/13	3/2	1.000
Tumor size (mm), median (range)	34.0 (17–58)	31.0 (22–63)	0.544
serum KL-6, median (range)	880.5 (325–2410)	544.0 (466–1199)	0.260
serum SP-D, median (range)	152.0 (60–502)	105.0 (31–140)	0.036
Respiratory function, median (range)			
%VC	90.7 (43.5–130.4)	86.7 (64.9–107.3)	0745
%FVC	81.8 (37.9–117.6)	82.7 (52.8–98.7)	0.820
FEV1.0 (L)	2.0 (1.13–2.92)	2.0 (1.47–2.96)	1.000
FEV1.0/VC (%)	80.7 (54.0–91.2)	78.6 (72.7–99.7)	0.696
%DLco	50.7 (15.5–92.2)	70.7 (39–235.5)	0.474
Not evaluated	3	0	
Dose-volume metrics, median (range)			
PTV (mL)	82.9 (34.6–345.9)	88.1 (52.3–1319.8)	0.507
Total dose (Gy[RBE]),	52.8 (46–72)	50.0 (50–72)	0.5745
Dosimetric factors of the lung (%), median (range)			
V5	8.7 (3.0–20.6)	14.2 (10.9–34.8)	0.026
V10	7.6 (2.6–18.3)	11.9 (8.4–32.8)	0.033
V15	5.6 (2.2–13.6)	8.0 (7.1–27.1)	0.074
V20	5.0 (3.0–20.6)	7.3 (6.2–18.0)	0.069
V25	4.2 (1.8–11.0)	5.4 (3.3–16.8)	0.237
V30	3.7 (1.5–9.0)	4.9 (2.8–15.9)	0.286
V35	3.4 (1.4–8.5)	4.4 (2.5–5.0)	0.260
V40	3.1 (1.3–8.0)	4.0 (2.2–14.0)	0.260
Mean Lung Dose (Gy[RBE])	2.8 (1.0–6.4)	3.8 (3.0–12.2)	0.075

*Abbreviations*: *CIRT* carbon ion radiotherapy, *ILD* interstitial lung disease, *JRS* Japanese Respiratory Society, *mMRC scale* the modified Medical Research Council scale, *KL-6* Krebs von den Lungen-6, *SP-D* surfactant protein D, *VC* vital capacity, *FVC* forced vital capacity, *FEV1.0* Forced Expiratory Volume in one second, *DLco* diffusion capacity of the lung, *PTV* planning target volume, *RBE* relative biological effectiveness

## Discussion

In this study, we aimed to evaluate the CIRT toxicity profile for LC patients with preexisting ILD-LC, for whom conventional radiation therapy such as SBRT is contraindicated. We evaluated 29 patients who were ineligible for surgery due to a previous diagnosis of ILD by examining the degree of RP in their lungs following treatment, as well as the degree of symptomatic change, and any AEs due to treatment.

SBRT is the typical first-line radiological intervention for early stage non-resectable LC. However, in the setting of ILD, the high risk of RP and AE limits radiotherapy to a small proportion of patients. In Yamashita et al.’s evaluation of SBRT in 117 ILD-LC patients, 9 cases of Grades 4–5 RP were noted and investigated; pretreatment blood KL-6 and SP-D levels, as well as the presence of an interstitial pneumonitis shadow, were found to be predictive of RP [25].

More recently, Ueki et al. evaluated 191 patients, 12 with mild and eight with severe cases of ILD. They found that the presence of ILD and the volume of the irradiated lung were associated with the development of grade 2 or higher RP. Furthermore, survival in the ILD group was poor compared to that in the base group [26]. Yamaguchi et al. further evaluated a group of 109 patients treated with SBRT at their institution. 16 were found to have subclinical ILD on CT and treated among these patients. Of 13 patients with post-treatment grades 2–5 RP, three were in the subclinical ILD group. Although subclinical ILD did not correlate with grades 2–5 RP in their study, the rate of extensive RP was notably high in these patients. Moreover, they reported that 2 patients with subclinical ILD developed Grade 4–5 RP [14].

The majority of the 29 patients in this trial presented with respiratory symptoms and clear interstitial opacities prior to treatment. Typically, this population of patients is offered best supportive care with no additional treatment. To our knowledge no other radiotherapy study targeted to this specific population has been performed.

The CTCAE were used to grade post-treatment pneumonitis. However, 14 of the 29 patients in our study (48%) presented with pretreatment grade 2-equivalent symptoms, while 4 (14%) presented with Grade 3 equivalent symptoms. As such, we postulate that the direct grading of symptoms following radiotherapy accuracy reflect post-treatment change. We evaluated the respiratory symptom grade progression over the course of treatment, and observed progression in 5 cases. Furthermore, ILD-related AE was noted in 2 patients; both grade 3 events. Despite the extremely high risk associated with these patients, the relative lack of serious adverse events (higher than grade 3) was noteworthy.

Although predicting the exact prognosis of ILD is difficult owing to the varying classifications involved, ILD-LC generally has a poorer prognosis in comparison to uncomplicated LC [4, 27]. In examining IPF specifically, Vancheri et al. and Ley et al. noted median survival times of between 2 and 3 years for IPF-LC patients [28, 29]. Prognosis times for ILD-LC patietns remain difficult to predict, especially for those diagnosed with ILD prior to developing LC and who therefore received prior treatment. In this study, approximately half of the patients (14) were discovered to have ILD at the time of their cancer diagnosis, while the remainder received an average of 4 years of treatment prior to their LC diagnosis.

Compared to surgical patients, the average age was high in our cohort, while forced vital capacity and diffusing capacity for carbon monoxide were low. Furthermore, the patients had clear interstitial changes on imaging that disqualified them from consideration for SBRT. Compared to patients who underwent SBRT, the rate of AEs in our patients was low, while comparable overall survival rates were noted. These data are shown in the Table 5 [5–9, 14, 30]. On the other hand, the local control rates in our study were lower than those among SBRT patients [14]. A possible explanation is that a higher percentage of our patients had T2 tumors (65.5%). However, the local control rates in patients without ILD in our study were comparable to those in patients who underwent SBRT ([16–19]), and patients with and without ILD underwent the same treatment protocol. Hence, further explanations remain elusive.

**Table 5:** Representative reported results of treatment for NSCLC with ILD

Author	year	treatment	disease	No of all pts	combined disease	No of pts. with ILD (%)	Age	median follow-up period (month)	AE (%)	Mortality after AE (%)	3 yr. OS (%)	5 yr. OS (%)	%FVC (or %VC)	%DLco
Chiyo	2003	surgery	NSCLC	931	ILD	36 (3.9)	65.5	43.8	9 (25)	1 (11.1)	41.5	35.6	84.9	―
Mizuno	2012	surgery	NSCLC	1444	IPF	62 (4.3)	71.6	―	7 (13.5)	6 (85.7)	―	―	%VC 95.3	51.1
Voltolini	2013	surgery	NSCLC	775	ILD	37 (4.8)	69.3	26	5 (13.5)	3 (60)	―	52	91.4	59.7
Sato	2014	surgery	NSCLC	41,742	ILD	1763 (4.2)	71	―	164 (9.3)	72 (43.9)	―	―	%VC < 80% 263	―
	2015										―	40	%VC > 80% 1478	―
Omori	2015	surgery	NSCLC	678	IPF	46	70.9	34.3	4 (8.7)	3 (75)	50.9	22.1	89.8	70.6
					Non-IPF	57	70.9		1 (1.8)	0	71.5	53.2	98.5	71.7
Yamaguchi	2013	SBRT	NSCLC, MLT	109	subclinical ILD	16 (14.7)	78	17.1	2^a^ (12.5)	1^b^ (50)	48	―	―	―
present	2015	CIRT	NSCLC	637	ILD	29 (4.6)	73	22.8	2 (6.9)	0	46.3	30.4	82.3	52.1
			stage I NSCLC	―	ILD	22	73	22.6	2 (9.1%)	0	57.2	42.1	81.8	55.1

^a^Grade4–5 RP, ^b^Grade5 RP

*Abbreviations*: *NSCLC* non-small cell lung cancer, *ILD* interstitial lung disease, *AE* acute exacerbation, *IPF* interstitial pulmonary fibrosis, *OS* overall survival rate, *FVC* forced vital capacity, *VC* vital capacity, *DLco* diffusion capacity of the lung, *MLT* metastatic lung tumor, *CIRT* carbon ion radiotherapy, *RP* radiation pneumotinis

In previous studies of LC treated with SBRT, PTV, V5,V13, V20, V25, Vprescription and mean lung dose were among the factors that showed a correlation with the severity of RP post-treatment [31–34]. In the 5 patients in our study who experienced disease progression, V5 and V10 lung doses were significantly associated with RP grade progression. However, the lung doses in these 5 patients are as low as the doses reported in the SBRT trials [13, 14, 33, 34], as heavy-ion therapy allows for improved minimization of non-target doses. Our results suggested that small differences in lung dose may significantly affect the severity of RP in symptomatic ILD patients.

With respect to the 2 cases that exhibited AE, we evaluated respiratory function tests, blood tests, dosimetric factors, and other factors to determine whether they predicted exacerbation. However, no such associations were found.

At PET pretreatment staging of the patients’ cancers, highly integrated mediastinal lymph nodes were noted in 3 cases, and an accessory nerve lymph node metastasis was noted in 1 case. Among these cases, a lymph node with a minor axis greater than 10 mm was found in only 1 patient. Roberts et al. previously reported that, among the 7 cases of mediastinal lymph node false positives of 100 examined on PET, 4 were likely caused by inflammation in the lung parenchyma [23]. Additionally, Konishi and colleagues examined 306 mediastinal lymph nodes in 54 patients and found 7 false positives, 2 of which were caused by interstitial pneumonia [22]. Considering these reports, the 4 cases of pretreatment lymph node metastases noted on PET/CT in our ILD-LC patients may have included false positives; hence, only the primary tumors were treated. Of the 4 potential metastasis cases, none were found to have lymph node metastasis after CIRT.

As this study was retrospective, there remains the possibility that selection bias may have impacted the results, as all patients included were ineligible for surgery and SBRT because of the clear risk posed by their ILD.

## Conclusions

In this study, most of patients presented with respiratory symptoms and clear interstitial opacities prior to treatment; this population had had few available treatment options outside of supportive care. Considering these unfavorable conditions, we demonstrated the efficacy and relative safety of the use of CIRT in LC patients with ILD. Dose-volume histogram analysis suggested the importance of minimizing the low-dose region, as the lung doses V5 and V10 lung doses were significantly associated with RP grade progression. As relatively few patients were evaluated in this study, a larger trial with long-term follow-up is warranted to verify these results.

## Abbreviations

^18^F–FDG: [18F]-fluorodeoxyglucose
AE: Acute exacerbation
CIRT: Carbon-ion radiotherapy
CT: Computed tomography
CTCAE: Common Terminology Criteria for Adverse Events
HIMAC: Heavy Ion Medical Accelerator in Chiba
ILD: Interstitial lung disease
IPF: Interstitial pulmonary fibrosis
KL-6: Krebs von den Lungen-6
LC: Lung cancer
LCR: Local control rate
MRI: Magnetic resonance imaging
NIRS: National Institute of Radiological Sciences
NSCLC: Non-small cell lung cancer
OS: Overall survival
PET: Positron emission tomography
PTV: Planning target volume
RBE: Relative biological effectiveness
RP: Radiation pneumonitis
SBRT: Stereotactic body radiation therapy
SP-D: Surfactant protein-D
